# An Electrochemical Sensor for the Detection of Albendazole Using Glassy Carbon Electrode Modified with Platinum-Palladium Nanocomposites

**DOI:** 10.3390/bios12111026

**Published:** 2022-11-16

**Authors:** Ghadeer A. R. Y. Suaifan, Mohammad F. Khanfar, Mayadah B. Shehadeh, Asmaa Alnajajrah, Raghad Abuhamdan, Sameer Ahmad Hasan

**Affiliations:** 1 Department of Pharmaceutical Sciences, Faculty of Pharmacy, The University of Jordan, Amman 11942, Jordan; 2 Pharmaceutical-Chemical Engineering Department, School of Applied Medical Sciences, German Jordanian University, P.O. Box 35247, Amman 11180, Jordan; 3 Department of Chemistry, The University of Jordan, Amman 11942, Jordan; 4 Biomedical Engineering Department, School of Applied Medical Sciences, German Jordanian University, P.O. Box 35247, Amman 11180, Jordan

**Keywords:** albendazole, cyclic voltammetry, differential pulse voltammetry, binary catalyst, electrode modification

## Abstract

An electroanalytical electrode for the detection of albendazole (ABZ) active ingredient in pharmaceutical dosage form and in contaminated animal-derived products was developed using a glassy carbon electrode modified with platinum-palladium nanoparticles. The electro-catalytic performance of the bimetallic-modified glassy carbon electrode was compared with its bare counterpart. Under optimized conditions, the modified electrode revealed two well-resolved anodic peak currents at 1.10 and 1.23 V using differential pulse voltammetry. Pure ABZ, as well as ABZ in spiked foods (milk and chicken), were detected with little interference from the food matrix. This electrode demonstrated high sensitivity and applicability, with a lower limit of detection of 0.08 µmol L^−1^ in aqueous solution and 10 µmol L^−1^ in the contaminated ground chicken and 100 µmol L^−1^ in the contaminated milk sample. The fabricated sensor is low in cost and appropriate for the estimation of albendazole in tablet dosage forms and biological samples, and so can act as a quality control tool in the pharmaceutical and food industry.

## 1. Introduction

**Scheme 1:**
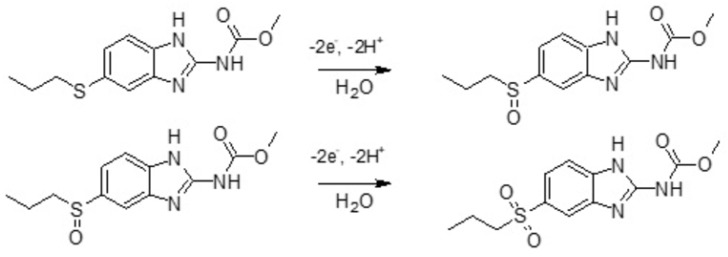
Proposed scheme for ABZ electrochemical oxidation.

Albendazole (Methyl [5-(propylthio)-1H-benzimidazole- 2-yl] carbamate) is a potent, broad-spectrum benzimidazole anthelmintic agent [1,2,3,4] used for the treatment of infections caused by intestinal helminths, such as *Echinococcus granulosus*, *Taenia solium* [5], *Enterobius vermicularis*, and *Trichuris trichiuria* [6]. Following oral administration, ABZ is transformed into its albendazole sulfoxide (ABZSO), albendazole sulfone (ABZSO_2_), and albendazole 2-aminosulfone (ABZ2-NH_2_SO_2_) metabolites (Scheme 1). ABZ anthelmintic activity is attributed to ABZSO main metabolite, which degrades helminths and larvae cytoplasmic microtubules [7]. Toxicological studies in animals have shown that ABZ and its active ABZSO metabolite are teratogenic, and thus may cause malformations and embryonic lethality [8,9]. The overuse of ABZ is a matter of concern and may harm global healthcare. Unfortunately, some breeders abuse the ABZ application in pursuit of greater economic benefits. Thus, large amounts of ABZ remain in animal-derived foods, which can pose health risks to consumers [8]. In the European Union, ABZ’s maximum residue limit in animal products has been stipulated to 100 μg/kg, based on the sum of ABZ and its three metabolites [10,11,12]. Therefore, ABZ detection is not only essential in monitoring the active ingredient and optimizing ABZ therapy in patients but also in tracing ABZ residue in animal products. Additionally, ABZ detection following its release from solid phase matrices is crucial in evaluating drug bioavailability [13].

Several techniques for the quantitative detection of ABZ active ingredient and pharmaceutical dosage forms have been commonly used, including titrimetry in non-aqueous medium and redox titrimetry [14,15,16,17], immunoassay [18], photometry [19,20], and liquid chromatography [21,22]. Although these assays are well-established, they require a long run time. Electroanalytical techniques should have attributes, such as quick response time, simplicity of use, cost-effectiveness, high sensitivity, selectivity for the determination of ABZ in different matrices, suitability for miniaturization, and portability, thus, enabling on-site monitoring [23]. In recent years, solid and modified electrodes were commonly used in electroanalytical measurement due to their ability in detecting drugs prone to undergoing oxidation reactions [24]. By taking measurements using cyclic voltammetry (CV), modified electrode sensitivity is kept track of. To perform the CV experiment, the choice of the working electrode is crucial [24]. Due to environmental concerns and EU regulations restricting the utilization and storage of mercury [25], the use of metallic (e.g., gold, platinum, silver, palladium, and indium tin oxide) and carbon-based (e.g., glassy carbon, carbon paste, boron doped diamond, and graphite) substrates has increased. Platinum-based electrodes have been widely used in electroanalyses, especially in anodic oxidation reactions where the analyte is electrolyzed at the platinum (Pt) surface in the presence of an auxiliary noble metal, such as palladium (Pd) or ruthenium, in a process known as the bi-functional mechanism. The oxidized form of the analyte then leaves the metallic surface with the generation of an oxidation current that could be correlated to the target analyte concentration [26,27,28,29]. For the detection of ABZ, electrochemical sensors have different advantages over classical methods, such as ease of sensors fabrication, significant sensitivity, and convenient usage. Interestingly, previous studies proved no effect for the presence or addition of various interfering agents, such as saccharine, sodium carbonate, citric acid, lactose, starch, and sodium bicarbonate, on ABZ voltametric signal [26,27,28]. In this work, attention has been paid to glassy carbon electrodes (GCEs), rather than its common analogue, the paste electrode, since GCE is more stable—not easily disintegrated.

ABZ electrochemical activity resides in its thio group that undergoes two consecutive oxidation steps, as shown in Scheme 1. The GCE has been used as a sensing platform in the recent electroanalytical field due to its reasonable conductivity, low-cost, good mechanical stability, ease of modification, and low background current [30,31]. In this work, we have studied the potential electrochemical detection of ABZ using Pt–Pd modified GCE. This study illustrates a new strategy through the rational design of simple, speedy, sensitive, and economically viable techniques for ABZ active pharmaceutical ingredient determination in pharmaceutical dosage forms and animal products.

Upon using voltage, ABZ goes through a two-step electro-oxidation reaction (Scheme 1). Electrolysis takes place at the thio group of the molecule. In the first step, the ABZ is converted to the corresponding sulfoxide, which is then transformed to the sulfone analogue, as shown in the scheme.

## 2. Experimental

### 2.1. Reagents and Chemicals

Glassy carbon electrode (PEEK isolated electrodes, 100 mm in length, 15 mm connector length, outer diameter = 6 mm, inner diameter = 3 mm), platinum reference, and Ag/AgCl counter electrodes (Fisherbrand™ accumet™ Glass Body—Mercury-Free) were purchased from Fisher Scientific, Greenville, NC, USA. Palladium (II) chloride, potassium tetrachloroplatinate (II), and Nafion were purchased from Sigma–Aldrich, Saint Louis, MA, USA. The carbon substrate, Vulcan XC72R, was a generous gift from Cabot Corp., Boston, MA, USA. Albendazole was a kind gift from the Jordanian Pharmaceutical Manufacturing Co., Amman, Jordan.

ABZ stock solution (1.0 µmol L^−1^) was freshly prepared in 1.0 mol L^−1^ HCl. The desired portions were withdrawn from the stock to prepare aqueous solutions with different electrolyte compositions and pH values: Britton–Robinson (BR) buffer (pH 2, 6 and 11) prepared by mixing 0.04 mol L^−1^ phosphoric acid, 0.04 mol L^−1^ boric acid, and 0.04 mol L^−1^ glacial acetic acids; phosphate buffer (pH 3) prepared by mixing Na_2_ HPO_4_, NaH_2_ PO_4_, and 0.5 mol L^−1^ H_2_SO_4_. The used chemicals were supplied by VWR Chemicals, PA, USA. All solutions were prepared with Millipore water, Milli-Q, MA, USA. The desired pH values were obtained by the addition of the appropriate amounts of 0.1 mol L^−1^ aq.NaOH solution. The pH values were measured using Hanna pH 211-microprocessor pH meter, Hanna Instruments, Inc., Woonsocket, RI, USA. The commercial ABZ drug was purchased from the local market.

### 2.2. Instruments

Impedance and voltammetric measurements (cyclic voltammetry—CV, and differential pulse voltammetry—DPV) were carried out in 100 mL three-compartment glass electrochemical cell utilizing platinum (0.25 cm^2^) and Ag/AgCl as the counter and reference electrodes, respectively. A glassy carbon disc electrode (3.0 mm i.d.) was used as the working electrode. Electrochemical measurements were performed by PGSTAT302 Autolab potentiostat (Metrohm, Utrecht, The Netherlands) controlled by NOVA 2.2 and connected to PC was utilized to execute the voltammetric experiments. The impedance results were obtained by using the FRA 32M module plugged in the potentiostat and the NOVA EIS data fitting software was used to fit the reported impedance results to the equivalent circuit. The metallic content of the black carbon catalyst was determined using thermogravimetric analysis using NETZSCH TG 209 Libra F1 analyzer, NETZSCH-Gerätebau GmbH, Germany. The 15,000× magnified image was obtained using Vega 3 scanning electron microscope Tescan, Czech Republic. All experiments were carried out at an ambient temperature of 25 °C.

### 2.3. Measurements and Procedure

Standard ABZ solutions were prepared from stock by serial dilution. The analytical curve, resulting from the prepared series, correlates the oxidation peak current to the corresponding analyte concentration. Each concentration was analyzed in triplicate. The voltammetric measurements were obtained using the differential pulse voltammetry with the following parameters: potential step 0.005 V, modulation amplitude 0.025 V, modulation time 0.05 s, interval time 0.5 s, at a scan rate of 0.010071 V/s. For ABZ determination, the potential of the modified electrode was scanned between 0.00 and 1.3 V versus Ag/AgCl.

### 2.4. Pt-Pd Catalyst Preparation and Glassy Carbon Electrode Modification

Platinum and palladium were chemically deposited as fine metals from their corresponding aqueous salts solutions. The salts were mixed with a high surface area carbon powder (Vulcan XC-72R) and the mixture was placed in a round bottom flask of a reflux system. Ascorbic acid, used as the reducing agent, was added to the flask via a dropping funnel at a constant rate (0.28 drop/s). The modified carbon powder was then separated from the reaction mixture and dried. An amount of 10 mg of the prepared powder was then mixed with 1.0 mL solution of ethanol:Nafion (700:300 μL) solution. The mixture was sonicated for 30 min to form an ink-like homogenous solution. An amount of 3 μL of the ink solution was dropped over working electrode, which had previously been polished with metallographic abrasive paper to remove the oxidation layer from the electrode sensing surface. The modified electrode was then left to dry in the air under ambient temperature. The catalyst was prepared with an equimolar amount of each metal. Nominally there should be equal amounts of the two metals.

### 2.5. Pharmaceutical Samples Preparation

A commercial sample of ABZ (200 mg per tablet) was purchased from the local market. Twenty tablets of the analyzed pharmaceutical formulation have been accurately weighed, finely ground, and transferred into a calibrated flask, which was completed to prepare 1.0 mmol L^−1^ aq.HCl solution to be equivalent to ABZ active ingredient in stock solution. Then, appropriate aliquots were diluted with BR buffer solution of the desired pH values. The percentage recovery was evaluated based on the following equation:

% recovery = ([(S_x + s_) − S_x_]/S_s_) × 100%

where S_x_ and S_s_ are the oxidation peak current values for the sample and the standard solutions, respectively, and S_x + s_ is the oxidation peak current for mixtures of equal volumes of the standard and the sample solutions.

### 2.6. Animal Products Preparation

The ground chicken sample was obtained from the local market. The sample was homogenized with Millipore water at 1:5 (w:v) ratio in a blender for 1 min and then rocked at room temperature for 2 h. Next, ABZ was contaminated in food matrices to create 10-fold dilution samples. The contaminated matrices were clarified by centrifugation (5 min, 2000 rpm) [32,33]. The supernatant was then collected and tested directly. Blank samples were contaminated with buffer and tested using the biosensor. Different ABZ concentrations 10^−3^–10^−6^ mol L^−1^ were prepared from ABZ (10^−3^ mmol L^−1^) stock solution. The experiments were conducted in triplicate.

Milk sample was purchased from a local supermarket. An amount of 1.0 mL of ABZ standard solution at the concentrations of 10^−3^–10^−6^ mol L^−1^ was added to 1.0 mL of milk samples, respectively. Next, the mixture was centrifuged at 10,000 rpm for 10 min at room temperature to discard some fat and precipitates, and the supernatant was analyzed directly. The analyses were repeated 3 times for each concentration.

## 3. Results and Discussion

### 3.1. Electrochemical Behavior of the Pt-Pd-Modified Glassy Carbon Electrode

**Figure 1:**
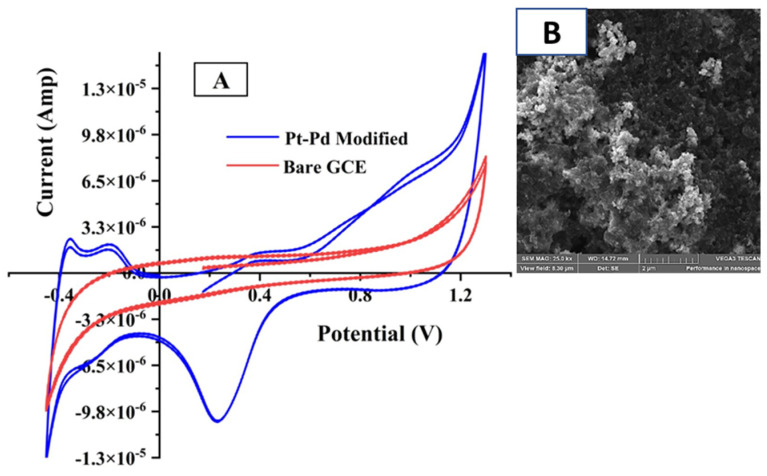
(**A**) Cyclic voltammograms of bare and of Pt-Pd-modified glassy carbon electrodes in BR buffer (pH 2.0). Scan rate = 50 mVs^−1^. (**B**) SEM image of Pt-Pd composite blended with black carbon.

Cyclic voltammograms of the bare and of Pt-Pd-modified glassy carbon electrodes in BR buffer (pH 2.0) are presented in Figure 1A. The bare electrode does not exhibit any specific voltammetric features over the scanned potential range. At potentials higher than 1.1 V, water oxidation commences while at voltages lower than −0.25 V, water reduction to molecular hydrogen takes place. At the modified surface, platinum oxide formation starts at potentials higher than 0.60 V, it lasts until the end of the anodic scan. When the scan direction is switched then the formed oxide is reduced back, generating a fresh platinum surface available for hydrogen adsorption at potentials lower than 0 V, under the utilized experimental conditions. The reported results confirm the presence of the noble metals as a layer over the modified glassy carbon surface. The presence of the bimetallic catalysts was verified by scanning electron microscopy. As shown in Figure 1B, the metallic composite is randomly distributed over the carbon surface, without any specific ordered shapes or any noticeable periodic repetition. The utilized catalyst preparation method is widely known to produce the bimetallic catalyst at the nanoscale [34,35,36]. The performed thermogravimetric analysis (Supplementary Materials), points to the presence of 20% metallic content of the loaded carbon catalyst. Yet further analysis is needed to define the stoichiometric ratio between the two deposited metals.

### 3.2. Effect of Different Electrolytes Compositions and pH Values on the Voltammetric Response of the Pt-Pd-Modified GCE

**Figure 2:**
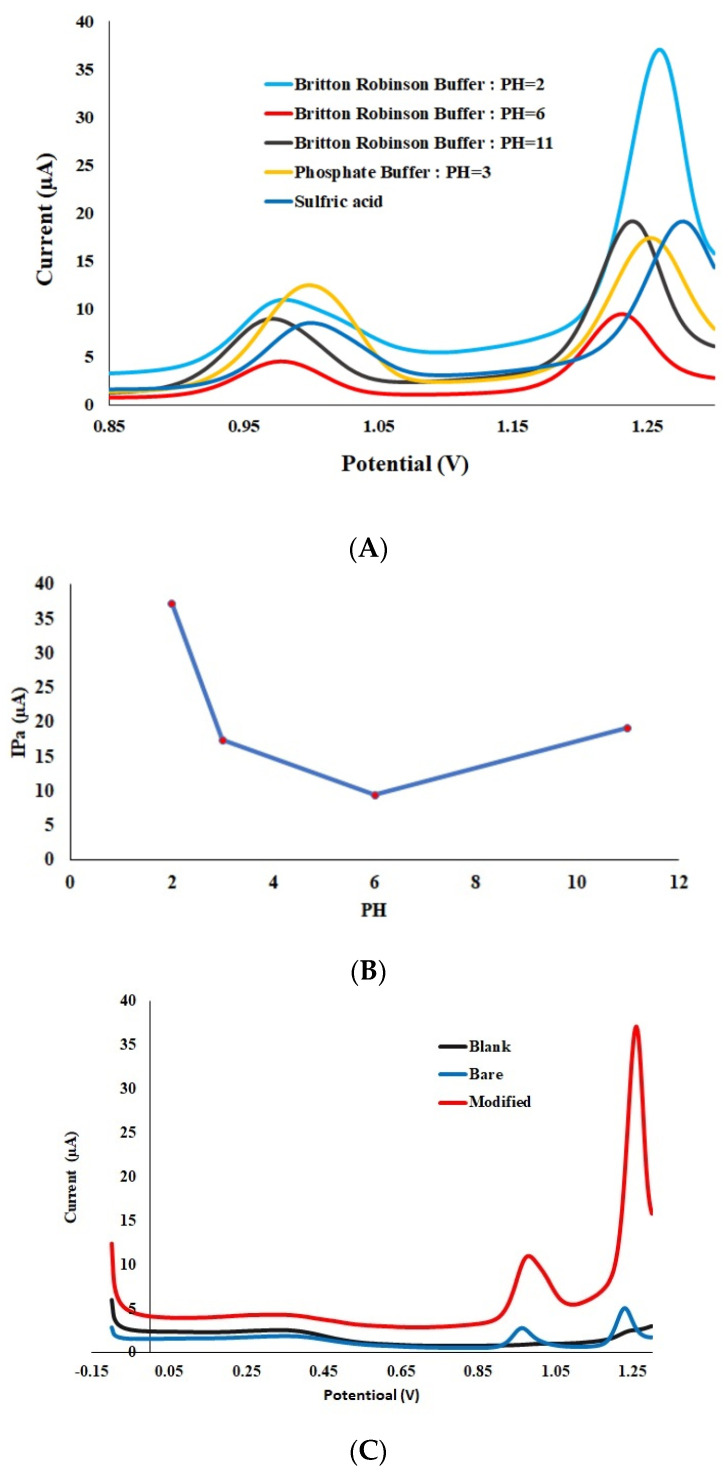
Voltammetric response of the modified electrode surface toward the oxidation of 1.0 × 10^−3^ M ABZ solutions. (**A**) Differential pulse voltammograms of Pt-Pd-modified GCE for ABZ solution at different buffer pH solutions. (**B**) Current variation of ABZ at modified GCE at different pH. (**C**) Differential pulse voltammograms of the bare and modified GCE in BR buffer solution containing 1.0 × 10^−3^ mol L^−1^ ABZ at pH 2.

Due to the low contribution of the charging current in DPV analysis to the total reported current, differential pulse voltammograms were utilized for ABZ analysis. Voltammetric response of the modified electrode surface toward the oxidation of ABZ was examined in aqueous solutions of different electrolytic compositions and pH values. As shown in Figure 2A, the optimum response was obtained when the potential of the modified electrode was scanned in BR buffer solution pH 2.0 at 25 °C. Consequently, BR buffer (pH 2.0) was selected for further investigations. The two peaks reported at 1.0 and 1.3 V could be attributed to the ABZ to sulfoxide, and the sulfoxide to sulfone electrochemical conversions, respectively, in the same manner shown in Scheme 1. Therefore, the reported data are consistent with the proposed scheme.

By elevation of the pH of the working solution, the corresponding oxidation peak current decreases, suggesting that protons actively participate in the mechanism of ABZ oxidation reaction. The number of electrons in the ABZ oxidation was estimated as equal to approximately two electrons for the first oxidation peak according to the CV theory for irreversible system [37] and using the following equation:

Ep/log ν = −30 mV/αn (1)

where Ep is the value of the peak potential, α the charge-transfer coefficient, n the number of electrons transferred, and v the scan rate. A linear relationship was observed for Ep vs. log ν in the range from 0.01 to 0.30 V s^−1^ for the first oxidation peak (1.25 V), with the slope equal to 35 mV. αn value was calculated as 1.16 from the obtained slope of the Ep vs. log v plots. Thus, using this information, assuming that α is equal to 0.5 (a value commonly assumed for organic compounds [38]), and using Equation (1), the number of electrons for the oxidation of ABZ was estimated. Figure 2B presents the plot of anodic oxidation peak current (Ipa) versus the pH of the utilized solutions. In this context, it is worth mentioning that ABZ solubility in aqueous solutions decreases as the solution’s acidity decreases. Another factor that affects the electrochemical behavior of ABZ is its acid dissociation constant, which is equal to 9.51 [39]. At pH values lower than its pKa, ABZ exists mainly in its most acidic form that is presented as the reactant in the first equation of Scheme 1.

The effect of modification of GCE by the Pt-Pd catalyst, on the oxidation of ABZ, is presented in Figure 2C. The oxidation current at the modified surface is three times higher than that reported at the bare electrode. The reported enhancement in the oxidation current could be attributed to the conductive nature and to the high surface area of the modifier, which enhances the charge transfer rate across the electrode–electrolyte interface when compared to its bare counterpart. The insertion of Pd to Pt catalysts enhances their catalytic activity, since the insertion process increases the catalyst surface area, reduces platinum poisoning, and resists variation in the bimetallic catalyst in acidic media [40]. In addition, palladium is much less expensive than platinum; therefore, the addition of palladium is of economic significance. The Pt-Pd catalysts are commonly utilized in fuel cells as catalysts; however, they have also been used for the detection of simple organic compounds of simple structures, such as glucose, cholesterol, hydrogen peroxide, and xanthine [41,42,43,44]. Under the utilized experimental conditions, the bimetallic catalyst is stable and does not undergo oxidation, as both Pd and Pt have oxidation potentials higher than all hydrocarbons, regardless of their nature as pharmaceuticals, natural products, artificial additives, and so on.

**Figure 3:**
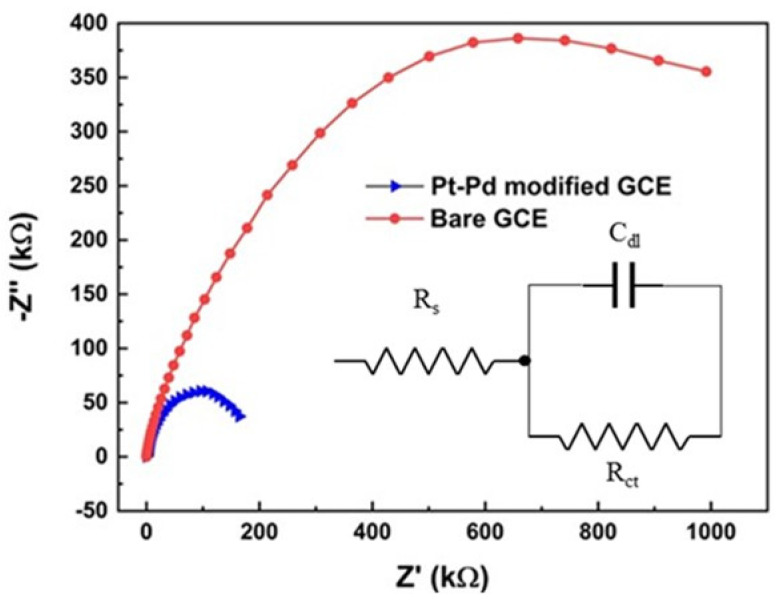
EIS Nyquist plots of the bare and the modified electrodes in BR buffer solution (pH 2). Rs: Solution resistance, Cdl: The capacitance of the electrical double layer at the electrode–solution interface, and Rct: The charge transfer resistance.

Enhancement of the working electrode conductivity, upon modification with the Pt-Pd layer, was verified by performing the corresponding electrochemical impedance spectroscopic (EIS) measurements, as shown in Figure 3, which presents the Nyquist plot of the modified and the bare electrodes in BRB pH 2 solution. The EIS Nyquist plots showed that the internal resistance of the Pt-Pd-modified GCE electrodes was about 52.8 Ω, which was less than that of the bare GCE electrode, where its internal resistance was 55.2 Ω. This is likely attributed to adding platinum and palladium metals to GCE. Moreover, the decrease in semicircle curve diameter highlights an improved electron transfer, thus, showing an increased conductivity. In addition, the maximum impedance value of the bare GCE was six times higher than that of the modified electrode. This suggests that the modified electrode has remarkably improved the working electrode conductivity and made the interfacial transfer much faster due to the high conductivity of Pt and Pd.

### 3.3. Effect of Scan Rate on ABZ Detection at the Pt-Pd GCE

The effect of the scan rate on the electro-oxidation of ABZ at the Pt-Pd-modified GCE was carried out by DPV analysis to obtain details about the ABZ oxidation manner from the relationship between the scan rate of the modified electrode potential and the corresponding peak current.

**Figure 4:**
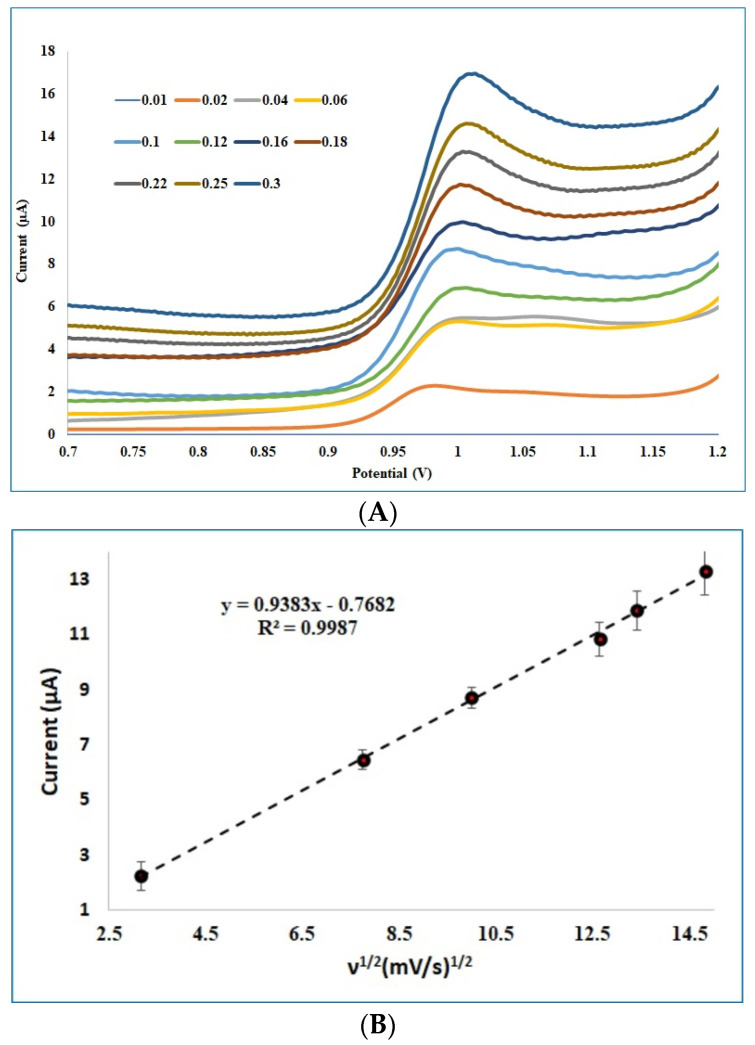
Electro-oxidation of ABZ at Pt-Pd-modified GCE. (**A**) Linear scan voltammograms of ABZ at different scan rates from 0.01 to 0.3 V s^−1^. (**B**) Dependence of the oxidation peak current on the square root of the scan rate.

The LSV of Pt-Pd GCE in 1.0 mmol L^−1^ ABZ (Figure 4A) was measured at different scan rates, varying from 0.01 to 0.30 V s^−1^. Peak current values were found to be linearly dependent on the square root of the scan rate. A linear correlation is investigated between the scan rate and the peak current (Figure 4B), indicating that the process is diffusion-controlled [30,31]. The corresponding regression equation is I (A) = −8×10^−7^ + 9×10^−7^ √ ν (V s^−1^); R^2^ = 0.9987. The scan rate values were selected to cover a reasonably wide range of scan rates from 5 to 300 mV/s. The values were selected so that the oxidation process could be detected at almost a constant potential ca 1.0 V with reasonable peak resolution.

### 3.4. Analytical Performance of ABZ Detection Using Pt-Pd-Modified GCE

**Figure 5:**
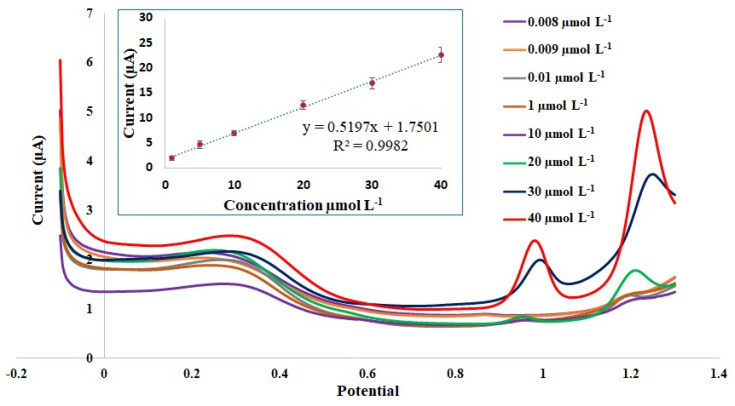
Dependence of the ABZ oxidation peaks on concentrations.

As shown in Scheme 1 and Figure 2A, ABZ exhibits two oxidation processes. All of the following obtained analytical data were withdrawn based on the first reported oxidation peak to eliminate any possibility of interference from any species that might be generated after the first oxidation process. Figure 5 shows the dependence of the ABZ oxidation peaks on concentrations. It is noted that the DPV oxidation signals increase gradually with the elevation in ABZ concentrations. A linear range was obtained from 3.12 × 10^−6^ to 2.26 × 10^−5^ mol L^−1^, I (A) = 0.51 [ABZ] (mol L^−1^) + 2× 10^−6^ (R^2^ = 0.998). Based on the signal-to-noise ratio of three trials, the detection limit was estimated at 8.18 × 10^−2^ µmoL L^−1^. Here, the signal-to-noise ratio was estimated based on the signal ratio between the blank and that of the ABZ which exhibits a three-fold increase in the instrumental signal. The reader must be aware of the fact that the corresponding lower limit of detection (LOD) value listed in Table 1 is of a different unit and magnitude and that modification was performed for matching purposes, so that all of the values listed in the table have similar units. In this work, a relatively high LOD was reported when compared to the corresponding values reported by the other research groups, as shown in Table 1. Further investigation is required to figure out the reasons behind the reported shortcoming.

The performance of the method utilized in this work could also be compared to that of other chromatographic methods employed for ABZ detection. Chromatography combines the efficiency of separation and the ability of detection. Table 1 demonstrates a comparison of the LOD in this study and the corresponding values obtained by other research groups. The Pt-Pd catalyst plays a significant role in the electron transfer rate enhancement, across the electrode–electrolyte interface, since it is more conductive than the corresponding bare GCE. Accordingly, the developed electrode exhibits a low limit of detection. Notably, the efficiency of the utilized electrode could be enhanced by the variation of thickness of the acting catalyst or by elevation of its metallic content, by either increasing its Pt and/or Pd proportion, or by the insertion of a third metal to the formulation, such as ruthenium, osmium, or iridium [4,57].

### 3.5. Determination of ABZ in Local Pharmaceuticals Dosage Form

**Figure 6:**
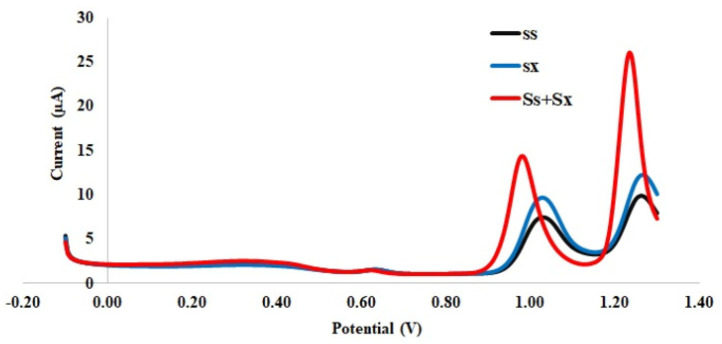
Differential pulse voltammograms of Pt-Pd-modified glassy carbon electrode A; in BR buffer pH solutions containing standard ABZ (S_s_), unknown ABZ (S_x_), and a mixture of the standard and the unknown (S_s + x_).

Recovery of the commercial medication was investigated in this work, as described in Section 2.5. One of the trials performed for estimation of the ABZ recovery, presented as an example, is shown in Figure 6. The variation in the position of the peak voltage is less than 25 mV. Thmole reported variation in the oxidation voltage values could be attributed to variations in the matrix of the analyte co-existing with other ingredients. A total of 85% recovery was obtained for the commercial formulation, which is within the acceptable range between 80 and 120%.

### 3.6. Detection of ABZ Contaminated in Animal Product Matrices

**Figure 7:**
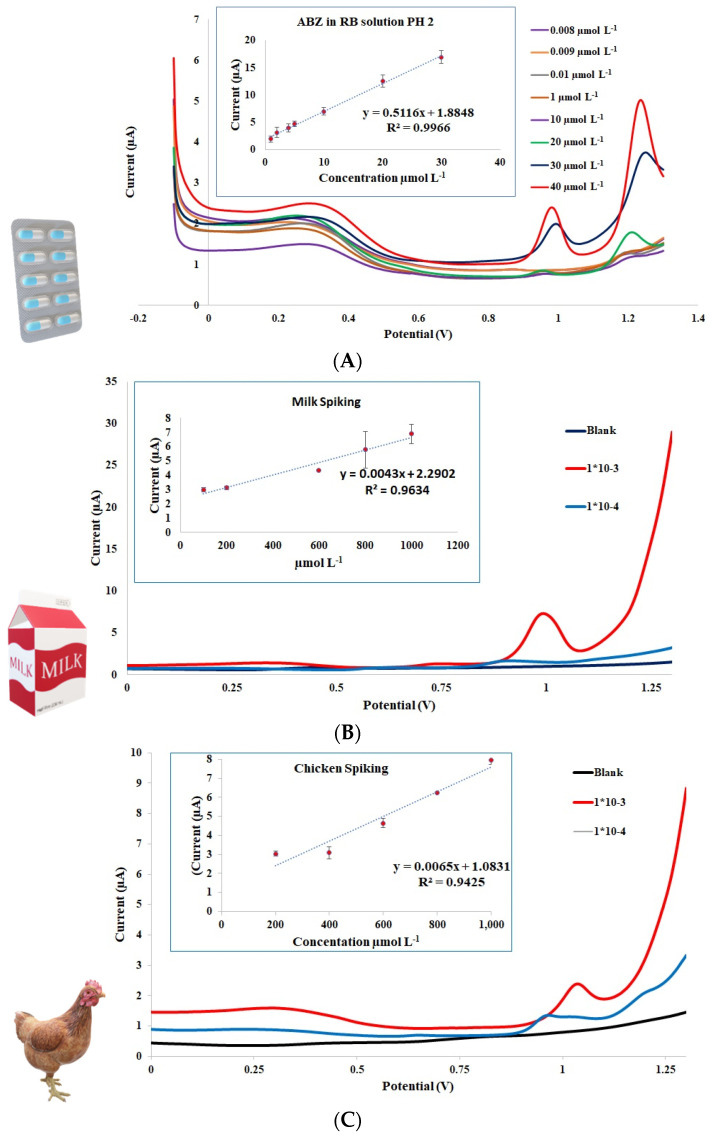
Application of Pt-Pd-modified GCE for ABZ detection. The plot of ABZ oxidation peak current versus its concentration in (**A**) BR buffer (pH 2); (**B**) in milk (pH 2); (**C**) in ground chicken (pH 2).

To determine Pt–Pd GCE platform feasibly for the detection of ABZ in contaminated animal-derived products, including ground chicken and milk, different concentrations of ABZ (range 10^−3^–10^−6^ mol L^−1^) were contaminated in the products. First, 1 mL of milk or ground chicken products were contaminated with 1.0 mL of ABZ at four concentrations, ranging from 10^−3^–10^−6^ mol L^−1^. A negative (blank) control was prepared by spiking food products with buffer only, containing no ABZ. The developed detection electrode displayed the ability to detect ABZ contamination in different food matrices, with a lower detection limit of 10^−4^ and 10^−5^ mol L^−1^ in milk and ground chicken, respectively, as shown in Figure 7, indicates that the Pt–Pd modified GCE can detect the target analyte effectively in contaminated animal products. Thus, the developed detection electrode would act as a quality control tool in the food industry due to its high sensitivity to detect low ABZ concentrations.

As shown in Figure 7, the disappearance of the second peak could be attributed to the matrix effect. One of the ingredients inherently co-existing with ABZ may interact with the sulfoxide product from the first electrolysis step (Scheme 1) and retards its oxidation at the modified sensor. In addition, adsorption of species from the investigated matrices at the sensor surface could not be excluded.

## 4. Conclusions

The current study describes the determination of ABZ by DPV technique, based on its oxidation over Pt-Pd-modified GCE. DPV technique gives a beneficial tool for the detection of low concentration levels of ABZ. The proposed methodology presented different advantages, such as simple catalyst preparation and easy GCE surface modification. This electrochemical electrode demonstrated high sensitivity and applicability, with lower limits of detection of 0.08 µmol L^−1^ in aqueous buffer solution, 10 µmol L^−1^ in the contaminated ground chicken sample, and 100 µmol L^−1^ in the contaminated milk sample, indicating that Pt-Pd-modified GCE can be used to trace the amount of ABZ in pharmaceutical formulations and contaminated animal-derived products, and thus can act as a quality control tool in the food industry. Additionally, reasonable recovery of ABZ analytes from a commercial product makes this method applicable in the clinical analysis. However, improvement in modified electrodes performance could be achieved by investigating the impact of variation of the catalyst layer thickness, the insertion of a third metal, and the composition of the materials binding to the metals, on the electro-catalytic activity of the utilized sensors toward the oxidation of ABZ. The investigation could be extended to include validation of the matching between the proposed mechanism of ABZ and the kinetic parameters of the oxidation process, such as the sensor surface area, the number of electrons transferred in the process, and the rate of ABZ diffusion toward the sensor surface.

## Figures and Tables

**Table 1:** Comparison of the activity of Pt-Pd/GCE for ABZ detection with other techniques.

Detection Method	LOD (μg mL^−1^) *	Reference
Titrimetric and spectrophotometric methods	0.237 μg mL^−1^ & 0.711 μg mL^−1^	[17]
Spectrophotometric	7.42 & 9.94 μg mL^−1^	[45]
Flow injection analysis (FIA) using UV detection & HPLC method	0.00318 μg mL^−1^ (FIA), 0.00297 μg mL^−1^ (HPLC)	[46]
Pectrophotometric (2 methods)	0.69 & 0.10 μg mL^−1^ 0.001438 μg mL^−1^	[47]
RP-HPLC–Mobile Phase (Methanol:acetonitrile:phosphate buffer (22:22:56))	3 ng mL^−1^	[48]
RP-HPLC–Mobile Phase (Acetonitrile:methanol:water (60:30:10))	N.A.	[49]
RP-HPLC–Mobile Phase (Acetonitrile:phosphate buffer (50:50))	0.001 μg mL^−1^	[50]
RP-HPLC–Mobile Phase (Acetonitrile:water:phosphate buffer (60:40:10))	0.0167 μg mL^−1^	[51]
Voltammetric method linear sweep (LSV), square-wave (SWV) and differential pulse voltammetry (DPV)	7.95 μg mL^−1^ (LSV), 16.45 μg mL^−1^ (SWV), 10.61 μg mL^−1^ (DPV)	[52]
Differential pulse cathodic stripping voltammetry (DPCSV) at the hanging drop mercury electrode (HMDE)	0.003 μg mL^−1^	[53]
Pencil graphite electrode Voltammetry	0.001438 μg mL^−1^	[54]
Modified sonogel with perovskite-type LaFeO_3_ nanoparticles	79.6 μg mL^−1^	[39]
Carbon paste electrode	0.056 μg mL^−1^	[55]
Cathodically pretreated at the boron doped diamond electrode	16.5 μg mL-1 (DPV), 43 μg mL^−1^ (SWV)	[56]
Electrochemical oxidation at the glassy carbon-rotating disk electrode	6.367 μg mL^−1^	[30]
**Current study**	**21.7 μg mL^−1^**	

* Values corrected for comparison purposes.

## Data Availability

Not applicable.

## Supplementary Materials

The following supporting information can be downloaded at: https://www.mdpi.com/article/10.3390/bios12111026/s1, Figure S1: Thermograph of Pt-Pd/Carbon catalyst. Heating rate = 10°C/min.
