# Supplementary material for: An Electrochemical Sensor for the Detection of Albendazole Using Glassy Carbon Electrode Modified with Platinum-Palladium Nanocomposites
Source: Biosensors (Basel). 2022 Nov 16;12(11):1026. doi: 10.3390/bios12111026 (PMC9688875; doi:10.3390/bios12111026)
Supplement: Supplementary file 1 [file biosensors-12-01026-s001.zip › biosensors-1979514-supplementary.pdf]

An Electrochemical Sensor for the Detection of Albendazole Using Glassy Carbon Electrode Modified with Platinum-Palladium Nanocomposites

Ghadeer A.R.Y Suaifan [1,*], Mohammad Khanfar [2], Mayadah B Shehadeh [1], Asmaa Alnajajrah [3], Raghad Abuhamdan [1] and Sameer Ahmad Hasan [4]

[1] Department of Pharmaceutical Sciences, Faculty of Pharmacy, The University of Jordan, Amman 11942, Jordan
[2] Pharmaceutical-Chemical Engineering Department, School of Applied Medical Sciences, German Jordanian University, P.O. Box 35247, Amman 11180, Jordan
[3] Department of Chemistry, The University of Jordan, Amman 11942, Jordan
[4] Biomedical Engineering Department, School of Applied Medical Sciences, German Jordanian University, P.O. Box 35247, Amman 11180, Jordan
* Correspondence: gh.suaifan@ju.edu.jo or ghadeer_petra@yahoo.com; Tel.: +962-7-7560-8695

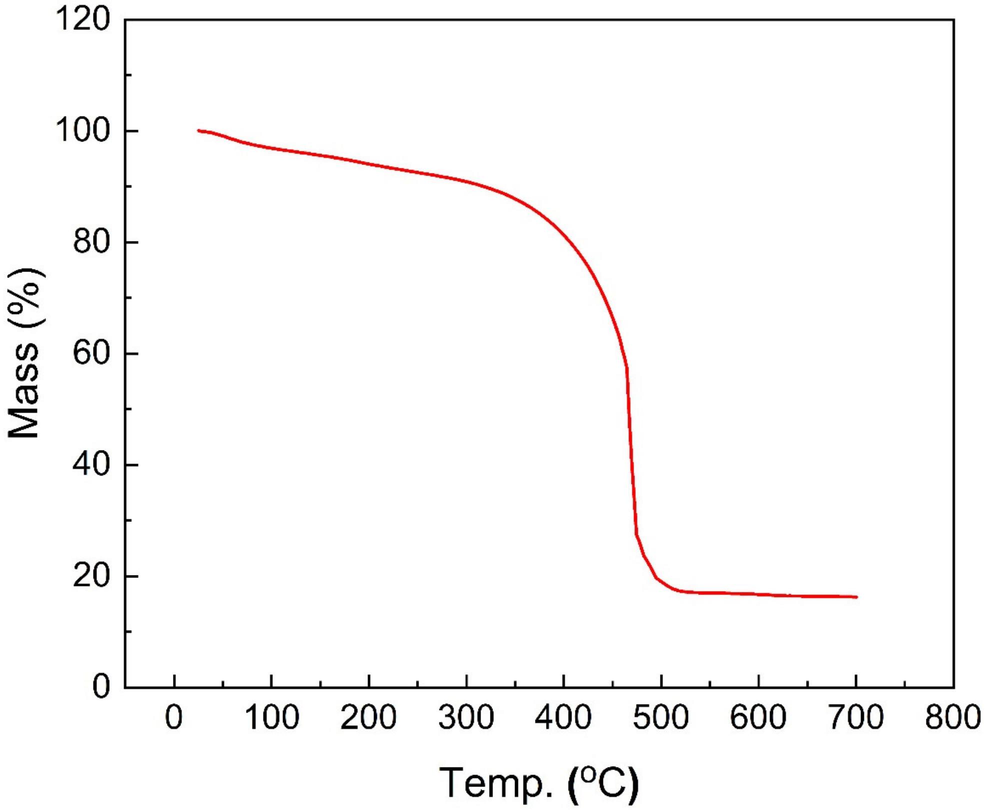

Figure S1. Thermograph of Pt-Pd/Carbon catalyst. Heating rate = 10 °C/min.
